# Enablers and Barriers to Nurse Managers' Leadership of Evidence‐Based Practice During Organisational Change: A Qualitative Study

**DOI:** 10.1002/nop2.70764

**Published:** 2026-08-13

**Authors:** Riitta Askola, Jaakko Varpula, Laura‐Maria Peltonen, Tarja Heino‐Tolonen, Miia Lindström, Anna Axelin

**Affiliations:** ^1^ Department of Nursing Science University of Turku Turku Finland; ^2^ Wellbeing Services County of Southwest Finland Turku Finland; ^3^ Department of Health and Social Management University of Eastern Finland Kuopio Finland

**Keywords:** evidence‐based practice, leadership, nurse managers, organisational change, qualitative research

## Abstract

**Aim:**

To explore the enablers and barriers to nurse managers' leadership of evidence‐based practice during organisational change, as perceived by clinical nurse specialists and nursing directors.

**Design:**

A qualitative descriptive interview study.

**Methods:**

Data were collected through semi‐structured interviews with clinical nurse specialists and nursing directors selected using purposive sampling. The data were analysed using inductive content analysis.

**Results:**

Three main categories were identified that influence nurse managers' leadership of evidence‐based practice during organisational change, as perceived by clinical nurse specialists and nursing directors: organisational readiness, partnerships and expertise in evidence‐based practice. Organisational readiness included standardised practices and supportive organisational structures. Partnerships involved collaboration with clinical nurse specialists, interprofessional teams and management. Expertise reflected nurse managers' competence in evidence‐based practice and the use of digital tools to assess care quality and effectiveness.

**Conclusion:**

Organisational readiness, partnerships and expertise in evidence‐based practice shape how nurse managers lead evidence‐based practice during organisational change. These factors influence the consistency of care, staff support and the ability to sustain evidence‐based practice in evolving healthcare environments.

**Implications for the Profession and/or Patient Care:**

Strengthening organisational readiness, building collaborative partnerships and supporting nurse managers' competence in leading evidence‐based practice can enhance care quality, staff wellbeing and patient outcomes during organisational change.

**Impact:**

This study addressed the challenge of sustaining evidence‐based practice during organisational change. The main findings highlight organisational, relational and competence‐related factors that influence nurse managers' ability to lead evidence‐based practice. The results are relevant for healthcare organisations, nurse leaders and policymakers seeking to strengthen leadership capacity and maintain evidence‐based practice during transitions.

**Reporting Method:**

This study adhered to relevant EQUATOR guidelines. The Standards for Reporting Qualitative Research (SRQR) checklist was used to guide reporting.

**Patient or Public Contribution:**

No patient or public contribution.

## 1 Introduction

Nurse managers play a pivotal role in driving successful organisational change, as they are uniquely positioned to coordinate processes, support staff and navigate the pressures created by large‐scale organisational change (Morrison and Jensen 2022). Across many countries, health care organisations are undergoing major structural changes that place increasing demands on leadership and the implementation of evidence‐based practice (EBP). In this context, nurse managers are central to creating organisational environments that support sustainable improvement and high‐quality patient care.

Successful implementation of EBP depends on supportive leadership, adequate organisational resources and an implementation climate that encourages learning and continuous improvement. Previous research has shown that strategic planning, resource allocation, leadership engagement and sustained managerial support are essential for embedding EBP into routine clinical practice (Aarons et al. 2024; Borge et al. 2022; Elsheikh et al. 2023). Both organisations and individual health care professionals must commit to using EBP in clinical decision‐making to achieve high‐quality care. Understanding how organisational support influences professional practice and patient outcomes is therefore essential for nurse managers leading EBP during organisational change (Ominyi et al. 2025). Without supportive organisational structures, nurses experience greater difficulty adopting evidence‐based interventions, limiting professional development, reducing adherence to best practices and ultimately affecting the quality of care (Huo et al. 2024; Kallio et al. 2024).

Although nurse managers' leadership of EBP has been widely studied, less is known about how this leadership is supported during periods of large‐scale organisational change. Existing research has focused primarily on nurse managers' own experiences, whereas the perspectives of clinical nurse specialists and nursing directors have received considerably less attention. Exploring these perspectives may contribute to a broader understanding of the organisational conditions that enable or hinder nurse managers' leadership of EBP during organisational change.

## 2 Background

Nurse managers have a central responsibility for implementing evidence‐based practice by creating supportive organisational cultures and facilitating the integration of research evidence into clinical care (Bianchi et al. 2018; Mathieson et al. 2019). Their role extends beyond encouraging the use of research evidence to supporting staff through change, ensuring access to education and resources, and addressing barriers that may hinder implementation (Guerrero et al. 2016).

Contemporary implementation research demonstrates that leadership behaviours, organisational support and implementation climate interact across multiple organisational levels to influence both the adoption and long‐term sustainment of EBP (Aarons et al. 2024; Williams et al. 2025). Effective leadership requires adequate education in EBP, sufficient organisational resources and sustained managerial engagement throughout the implementation process (Mathew et al. 2024). Leadership for EBP is therefore distributed across multiple levels of the health care system.

At the micro level, nurse managers facilitate the use of research evidence by organising education, supporting staff and encouraging evidence‐informed clinical decision‐making. Clinical nurse specialists complement this role by mentoring staff, providing expert clinical guidance and supporting the implementation of evidence‐based interventions in everyday practice. Nursing directors, while less involved in direct patient care, create strategic conditions for EBP through resource allocation and by fostering organisational cultures that value evidence‐informed decision‐making (Melnyk and Fineout‐Overholt 2018).

At the meso level, organisational factors strongly influence nurses' ability to implement evidence in practice. Policy‐relevant strategies include formalising protected time, resourcing embedded facilitation, investing in knowledge infrastructure and expanding clinical academic pathways to create environments where evidence use becomes routine and supported (Ominyi et al. 2025). Nurse managers are instrumental in establishing organisational structures and learning cultures that support evidence integration (Furtado et al. 2024). Clinical nurse specialists often coordinate EBP initiatives, contribute to clinical guideline development and act as intermediaries between frontline clinicians and management (Atalla et al. 2025; Hako et al. 2023). Nursing directors provide strategic leadership for EBP implementation, while nurse managers operationalise these strategies within clinical units (Sebire et al. 2025).

At the macro level, national policies, legislation, funding models and educational frameworks shape the implementation of evidence‐based nursing (WHO 2020). Nurse managers, clinical nurse specialists, and nursing directors may contribute to national guideline development, professional networks, and policy initiatives that strengthen evidence‐informed health care. Leadership for EBP therefore relies on collaboration across organisational and system levels rather than on the actions of individual leaders alone (Harvey et al. 2019).

Health care systems worldwide are currently undergoing substantial organisational reforms, creating new expectations for leadership competencies and workforce capabilities (Fagerström 2021). In Finland, a major health and social services reform was implemented in 2023, transferring responsibility for health, social welfare and rescue services from municipalities to 22 larger organisations. The reform aims to improve equality, cost‐effectiveness, collaboration and knowledge‐based management (Ministry of Social Affairs and Health 2024). At the same time, widespread workforce shortages continue to challenge the availability of health and social care services (THL 2023). Despite these pressures, implementing evidence‐based care remains both a legal requirement under the Health Care Act (1326/ 2010) and the Act on the Status and Rights of Patients (785/ 1992) and an essential component of safe, high‐quality care.

Taken together, previous research provides limited insight into how organisational change influences support for nurse managers' leadership of EBP from the perspective of their key collaborators. Examining the perspectives of clinical nurse specialists and nursing directors may therefore provide a more comprehensive understanding of how EBP leadership is enacted and sustained during organisational change.

## 3 The Study

The aim of this study is to explore the enablers and barriers to nurse managers' leadership of evidence‐based practice during organisational change, as perceived by clinical nurse specialists and nursing directors. The findings may contribute to a broader understanding of how organisational conditions influence nurse managers' leadership of evidence‐based practice during periods of organisational change.

## 4 Methods

### 4.1 Design

A qualitative descriptive interview study design was used.

### 4.2 Setting and Recruitment

This study was conducted in one of Finland's largest wellbeing services counties which was established in January 2023. The county has a population of around 500,000 inhabitants and is one of Finland's seven bilingual wellbeing services counties. It is responsible for hospital services, social welfare and health care services, services for the elderly and rescue services. The county comprises over 25 regional municipalities and employs more than 20,000 people. Of these, more than 500 are health care managers with varying levels of education. Some individuals hold a university degree, while others possess a nursing degree combined with extensive work experience. The scope of this study comprises social welfare and health care services, hospital services and services for the elderly.

Purposive sampling was selected to gather in‐depth insights from individuals who could provide a diverse perspective on EBP in health care, which has been scarcely studied from their point of view. Clinical nurse specialists and nursing directors were purposefully selected as informants due to their strategic roles in supporting nurse managers in leading EBPs.

### 4.3 Inclusion and/or Exclusion Criteria

The inclusion criteria were as follows: (1) working as a nursing director or clinical nurse specialist during the organisational change, (2) having direct responsibility for leading or supporting evidence‐based practice and (3) being able to describe their experiences of the implementation process. Individuals who did not hold a managerial or specialist role during the change were excluded. As advanced‐practice nurses with a master's, doctoral or post graduate certificate obtained in a clinical nurse specialist programme (National Association of Clinical Nurse Specialists 2024), clinical nurse specialists offer clinical expertise and a perspective that has been underrepresented in implementation research (Crawford et al. 2023; Jokiniemi et al. 2023; Siedlecki 2026). Nursing directors strategize, align goals, oversee operations and ensure adequate resources for nursing (Kelly et al. 2023), thus being pivotal in enabling the management of EBP. In this study, both groups assess the role and actions of nurse managers in leading EBP during a period of organisational change. Their insights are essential for understanding how leadership at different levels influences the successful integration of EBP in evolving health care environments. To ensure variation, participants were selected from different service areas, including social welfare and health care services, hospital services and services for older people. They also represented a range of managerial experience—from early‐career to senior managers—and organisational units of different sizes. This heterogeneity enabled the sample to capture diverse perspectives on the leadership of EBP across settings.

### 4.4 Data Collection

Recruitment was initiated by approaching two executive managers from the research organisation. They provided a list of clinical nurse specialists considered suitable informants because they had comprehensive knowledge of the phenomenon. The researchers then compiled a list of nursing directors from social welfare and health care services, hospital services and services for the elderly. The first researcher (R.A.) sent an email with information about the study and an invitation to participate to 21 individuals, seven of whom agreed to participate in the study. The executive managers were unsure whether a particular individual had participated or not.

The research team considered a sample of seven interviewees to be sufficient for this qualitative study, as the research was narrowly focused and context‐specific. Although the number of participants was limited (*n* = 7), this aligns with qualitative research approaches that prioritise depth of understanding and rich, context‐specific insights (Guest et al. 2020; Saunders et al. 2018; Vasileiou et al. 2018). The adequacy of the sample size was assessed throughout both data collection and analysis. Because the study focused on a clearly defined phenomenon and the participants were directly involved in the organisational change, each interview generated rich, experience‐based insights. Since the same researcher conducted all interviews, recurring themes could be identified during data collection. As the interviews progressed, similar perspectives and examples began to recur, indicating that additional interviews were unlikely to yield new information. No new codes or categories emerged after the sixth interview, and the seventh interview confirmed the stability of the findings. Based on this assessment of data saturation, the final sample of seven participants was considered sufficient (Holloway and Wheeler 2010; Saunders et al. 2018). This conclusion was further supported through discussions within the research group, after which data collection was discontinued.

Semi‐structured interviews were conducted with clinical nurse specialists and nursing directors to explore their perspectives on how nurse managers lead the implementation of evidence‐based practices, how existing structures and environments support or hinder nurse managers in leading EBP, how they evaluate the effectiveness of EBP, and what information and support nurse managers need to effectively lead EBP. In addition, follow‐up questions were asked as needed. The interview guide (Table 1), developed by the researchers, was not pilot‐tested as its clarity and relevance were deemed sufficient.

**TABLE 1:** The interview guide.

What are the conditions that enable nurse managers to lead evidence‐based practice, and how, in your view, do nurse managers lead these practices?
2 How do nurse managers lead the implementation of evidence‐based practice?
2.1 What is your role in the implementation of evidence‐based practice?
2.2 What is your collaboration with nurse managers like?
3 How do existing organisational structures and the work environment support or hinder nurse managers' leadership of evidence‐based practice?
3.1 How would you develop the structures or environment to better support nurse managers' leadership of evidence‐based practice?
4 How do nurse managers evaluate the effectiveness of evidence‐based practice?
4.1 What is your role in evaluating the effectiveness of evidence‐based practice?
4.2 How would you improve the evaluation of the effectiveness of evidence‐based practice?
4.3 What impact does the leadership of evidence‐based practice have?
5 What knowledge and support needs do nurse managers have for leading evidence‐based practice?
5.1 What kind of knowledge and support should be provided, and how should it be organised?

Data were gathered through semi‐structured individual interviews with clinical nurse specialists (*N* = 4) and nursing directors (*N* = 3). The interviews were conducted between September and December 2024 via Microsoft Teams. One interviewer (R.A.) or, in some cases, two interviewers (R.A. and J.V.) were present during the interviews. The interviews lasted on average 1–1½ hours and were recorded and transcribed. In total, the data consisted of approximately 8.5 h and 180 pages (1.5 spacing) of interview material. During the transcription of the interviews, participants were deidentified to ensure their confidentiality and anonymity. Any statements or comments that could potentially reveal their identities or compromise their privacy were omitted without altering the content of the interviews. The interview transcripts were not returned to participants for review, as the analysis focused on the content produced during the interview itself rather than on retrospective revisions.

### 4.5 Data Analysis

**FIGURE 1:**
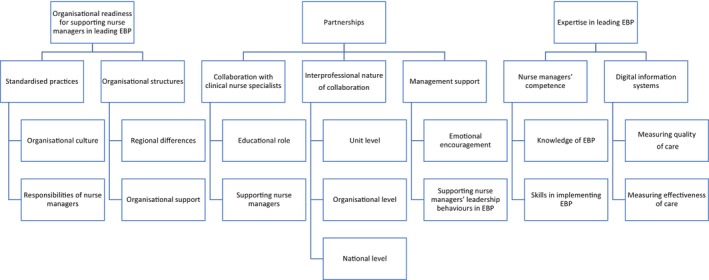
Summary of categories.

The data were analysed by one researcher (R.A.) using inductive content analysis, which was chosen because the researchers wanted to describe and understand the human experiences and perspectives of the phenomenon under study without predefined categories or a pre‐existing theoretical framework (Kyngäs 2020). The data were organised using the NVivo coding assistance software. The researcher (R.A.) reviewed the transcripts multiple times to gain a comprehensive understanding of the text. A theme relevant to the topic was chosen as the unit of analysis. The data were organised through open coding, coding sheets, grouping, categorisation and abstraction (Elo and Kyngäs 2008). The researcher (R.A.) identified sentences and paragraphs that thematically aligned with the research question. The coded data were then condensed, with comparisons and groupings based on similarities, creating subcategories with similar expressions. The saturation of categories was reached as no additional categories emerged, clarifying the relationships between codes and the identified themes. Ultimately, the researcher (R.A.) developed upper categories and main categories, which were derived from these subcategories (Figure 1). The results were initially analysed by one researcher (R.A.), then discussed with a second researcher (A.A.) and finally agreed upon by the research group. The views of the clinical nurse specialists and nursing directors did not differ from each other and could be combined.

### 4.6 Ethical Considerations

Ethical permission for the study (TY/461/06.01.01/2024) was reviewed by The Health Care Division of the Ethics Committee for Human Sciences at the University of Turku, and permission for the study was granted by the research organisation. All informants were provided with information about the study and were assured of anonymity and confidentiality in their reporting. They signed an informed written consent form. No demographic information was collected from the participants to preserve their anonymity.

### 4.7 Rigour and Reflexivity

The rigour of the analysis was strengthened through the criteria of credibility, dependability, transferability and confirmability (Graneheim and Lundman 2004; Guba and Lincoln 1985). Informants were selected for their diverse, in‐depth knowledge. During the interviews, the researcher (R.A.) asked follow‐up questions to ensure accurate interpretations and to reflect the participants' lived experiences. The researcher aimed to accurately portray the participants using their direct expressions. The goal of the content analysis was to simplify the material and create categories that reliably represented the original data. The informants' experiences were extracted as thick descriptions of the study phenomenon. The researcher (R.A.) revisited the original material to ensure the categories truly reflected it. The researcher's initial understanding played a crucial role in the production, description and interpretation of data, facilitating an understanding of the interviewees' experiences, perceptions and viewpoints, thereby enhancing the trustworthiness of the study. A detailed contextual description of the research context, participants and methods was provided. The results outline the analysis conducted, along with the study's strengths and limitations.

The research team consisted of university‐based researchers with expertise in qualitative methods and evidence‐based practice, as well as senior nursing leaders from the participating organisation. The involvement of senior leaders provided contextual knowledge of the organisational structures and ongoing change processes, while the academic researchers contributed methodological rigour and an external perspective. None of the researchers had a supervisory relationship with the participants, and no pre‐existing personal relationships were present, which helped minimise role‐related bias during data collection.

Because data were collected from clinical nurse specialists and nursing directors—rather than nurse managers themselves—the findings reflect second‐order interpretations of nurse managers' leadership of evidence‐based practice. This may introduce interpretive distance, as participants described their perceptions of nurse managers' actions rather than their own lived experiences. This potential limitation was acknowledged during analysis, and interpretations were grounded in verbatim data to remain close to participants' accounts.

## 5 Findings

### 5.1 Participants

The study included seven participants: four clinical nurse specialists and three nursing directors. Together, they represented key professional groups working closely with nurse managers and contributed complementary perspectives on the organisational conditions shaping the leadership of EBP during organisational change. The nursing directors worked across both social welfare and health care services and hospital services, while the clinical nurse specialists represented different organisational sectors, ensuring diversity of operational contexts without revealing individual roles.

In this study, three main categories emerged from the perspectives of clinical nurse specialists and nursing directors regarding the enablers and barriers for nurse managers in leading EBP during an organisational change. These main categories were (1) organisational readiness for supporting nurse managers in leading EBP, (2) partnerships and (3) expertise in leading EBP. Each main category consisted of upper categories followed by sub‐categories of related sections (Figure 1).

### 5.2 Organisational Readiness for Supporting Nurse Managers in Leading Evidence‐Based Practice

According to the clinical nurse specialists and nursing directors, nurse managers' abilities to lead evidence‐based practice during organisational change were influenced by the organisation's readiness to support them in their leadership of EBP. This main category comprised two upper categories: (1) standardised practices during the change and (2) organisational structures.

#### 5.2.1 Standardised Practices During Change

According to the clinical nurse specialists and nursing directors, standardised practices were essential enablers for nurse managers in leading EBP during organisational change. These practices were associated with the organisational culture and the responsibilities of nurse managers.

According to clinical nurse specialists and nursing directors, an essential enabler for nurse managers in leading EBP was an organisational culture that adequately supported nurse managers in promoting EBP. Clinical nurse specialists and nursing directors recognised that ongoing organisational change revealed the prevalence of outdated, evidence‐lacking practices, and that knowledge of EBP and their significance was not widely understood or established. This created a setting in which EBP was valued, supported and expected unevenly, thereby hindering nurse managers' capacities to lead EBP. The model and recommendations for evidence‐based practice were not uniformly known or applied across different units. Respondents observed that the implementation process of EBP was affected by cultural changes, lingering old practices and a lack of coherent leadership, as the organisational change had not led to a stable and consistent approach. As a result, the practices for leading evidence‐based practice were inconsistent. Additionally, a high turnover rate among nurses negatively impacted the implementation process, as onboarding new staff and ensuring they learn the required practices demanded significant time and resources.

When there is such a huge organisational change, it forces a new perspective,—and then it has to be rebuilt. P4

According to some respondents, the organisational change significantly impacted the responsibilities of nurse managers to the extent that it undermined the management of evidence‐based practice. They pointed out that while nurse managers wanted to develop their work, they lacked the opportunity due to their heavy workloads. This increased workload caused by the organisational change was attributed to a rise in staffing levels and a lack of clarity in the job descriptions for nurse managers. Respondents observed that nurse managers were overly focused on human resource management tasks, often performing duties that were not part of their responsibilities.

At the moment, these changes and circumstances have made it less visible what the nurse managers have actually been able to achieve in terms of evidence‐based nursing. P1

#### 5.2.2 Organisational Structures

According to the clinical nurse specialists and nursing directors, organisational structures emerged as a key enabler for nurse managers in leading EBP during organisational change. Their effectiveness was closely linked to recognising regional differences and ensuring adequate organisational support.

According to the clinical nurse specialists and nursing directors, straightforward and process‐oriented organisational structures had a significant impact on the ability of nurse managers to lead evidence‐based practice during organisational change. In the context of organisational change and the integration of regional municipalities, it was essential to provide equal opportunities for specialised and primary care and to identify regional differences that impact the ability of nurse managers to lead EBP successfully. In the wellbeing services county under study, the uneven distribution of clinical nurse specialist resources across different services was identified as a barrier for nurse managers in leading EBP. Additionally, specialised care and primary care were perceived as being in an unequal position. Moreover, quality management platforms and integrated management systems that support nurse managers in leading EBP were not used consistently across the organisation.

Archipelago regions, the Swedish‐speaking area, are vulnerable precisely because of their small size…you have to have that well‐trained reserve. Where do you get the employees so that the level or quality of care does not vary too much? P1

The influence of organisational support, including low organisational bureaucracy, sufficient resources, adequate staffing, clear communication and information, enabled nurse managers to lead EBP. Respondents emphasised the importance of having enough staff and strong nursing leadership to outline nursing strategy and goals. Well‐organised Human Resource support services were seen as a way to free up nurse managers' time to lead EBP.

Heavy bureaucracies challenge leadership here, especially the management of evidence‐based nursing. P2

### 5.3 Partnerships

Multilevel partnership was emphasised as an essential enabler for nurse managers in leading EBP during organisational change. Three upper categories emerged from the participants' descriptions regarding elements of ‘Partnerships’. These categories are: (1) collaboration with the clinical nurse specialists, (2) interprofessional nature of collaboration and (3) management support.

#### 5.3.1 Collaboration With Clinical Nurse Specialists

According to the clinical nurse specialists and nursing directors, the organisational change led many nurse managers to adopt a new way of working with clinical nurse specialists, despite unevenly distributed resources. The role of the specialists was crucial in empowering nurse managers in leading EBP. Their responsibilities included educating staff on these practices and supporting nurse managers in their implementation. Respondents emphasised the vital role of clinical nurse specialists in designing and delivering staff education. Their role was particularly important in synthesising and transferring EBP according to the framework provided by the organisation. The clinical nurse specialists actively sought out this information both nationally and internationally.

The aim is to establish consistent practices within our services. P3

According to the respondents, clinical nurse specialists play an essential role in supporting and motivating nurse managers in leading and implementing EBP, particularly in advancing projects and initiatives. They lead nursing development workgroups and collaborate with relevant stakeholders. Although their role is defined as expert‐based without administrative duties, their responsibilities and scope of duties remain somewhat unclear.

If the need for development arises from the nurse manager level, they usually also recognize its benefits and other implications. When a clinical nurse specialist is brought in for support, the two of them can often manage the process together quite effectively. P5

#### 5.3.2 Interprofessional Nature of Collaboration

The clinical nurse specialists and the nursing directors emphasised the importance of interprofessional collaboration in supporting nurse managers in leading EBP and the need for such efforts at the unit, organisational and national levels.

At the unit level, nurse networks and clinical nurse specialists played a crucial role in implementing EBP. The collaboration had increased in several services during and after the organisational change, as reflected in the increase in consultations between nurses across units. Responses highlighted the importance of clinical nurse specialists' role in promoting and working with the latest research evidence. In addition, the role of assistant nurse managers was expected to be strengthened to support the nurse manager in leading EBP, and it was hoped that their role would be further strengthened within the organisation.

We have recognized the assistant nurse managers' role as very, very central, and we are concerned that they are not, for example, in the nurse manager meetings where these matters are discussed. P3

The respondents described that at the organisational level, interprofessional collaboration emphasised the importance of expertise and understanding in EBP. Interprofessional collaboration and consultation occurred across various professional groups and medical specialisations, supporting the implementation of evidence‐based practice. This integrated approach facilitated the sharing of expertise and promoted consistent, high‐quality clinical decision‐making aligned with current best practices. It was also important to ensure that the client's perspective was taken into account and, through their feedback, highlight the effectiveness and health benefits of the treatment

We need to include the service users in this, meaning even more strongly the clients and patients. P4

According to the clinical nurse specialists and nursing directors, networking at the national level was seen as a critical resource for promoting evidence‐based practice, as practices should be standardised across the wellbeing services counties. This was seen as a matter of coordination across various areas, utilising evidence‐based structures and practices for developing, verifying and monitoring competence, promoting regional cooperation between operating units and training organisations, and encouraging multi‐professional development of management skills. This approach has the potential to create harmonised strategies that would significantly enhance nurse managers' ability to lead EBP. This networking occurred with the Nursing Research Center, various universities and other health‐related organisations.

These large‐scale national development projects initiated by the Ministry of Social Affairs and Health, including major ongoing initiatives we are involved in, are running in parallel and have a significant impact on other areas of our work as well. P4

#### 5.3.3 Management Support

According to the clinical nurse specialists and nursing directors, management support was essential to help nurse managers navigate the change process and establish the structure necessary for EBP. This support included both emotional encouragement and concretely supporting the nurse managers' leadership behaviours in EBP.

The nursing directors played a critical role in emotionally encouraging the nurse managers in leading evidence‐based practice. Both the clinical nurse specialists and nursing directors provided support by reminding, communicating and negotiating with nurse managers. The organisational change and new practices, including the implementation of EBP, had caused nurse managers to face resistance from the staff.

Their role in implementing it in the field has not been easy. My own role has been to support and encourage them in this process, acknowledging that there is often resistance to change. P6

To support nurse managers in leading evidence‐based practice, they also needed support in their leadership behaviours. The organisational change required nurse managers to adopt new roles and responsibilities, which presented challenges in adjusting to these expanded expectations. This transition affected their level of self‐direction, as they were not as autonomous in their decision‐making as had been anticipated. Furthermore, many inexperienced nurse managers required assistance with their decision‐making processes, time management and methods for leading EBP.

If all nurse managers had received sufficient information about the change process, it could have made their work easier overall. Having that knowledge and understanding—that we were actually going through a change—might have made it easier to guide evidence‐based practice forward. With awareness of the change process and its phases, it would have been easier to lead in line with it, at least in theory. P1

### 5.4 Expertise in Leading Evidence‐Based Practice

The two upper categories under ‘Expertise in leading EBP’ were categorised, as per the participants' descriptions, as elements of expertise in leading EBP that affected the nurse managers' ability to lead evidence‐based practice in a changing organisation. These were (1) nurse managers' competence and (2) digital information systems for measuring the quality and effectiveness of care.

#### 5.4.1 Nurse Managers' Competence

According to the clinical nurse specialists and nursing directors, having sufficient competence to lead EBP is critical for a nurse manager. This competence is viewed as encompassing knowledge of EBP principles and skills in implementing EBP.

During the organisational change, the organisation had nurse managers with different educational backgrounds, which affected their ability to lead EBP. Respondents observed differences in the knowledge of nurse managers and largely agreed that the education of nurse managers, especially at universities and universities of applied sciences, had provided a strong foundation in knowledge of evidence‐based practice principles. Some respondents also highlighted that leading EBP also requires sufficient work experience in nurse management.

It is so important that they all have some kind of education and training. P6

The organisational change underscored the importance of nurse managers possessing prerequisite skills specifically related to the implementation of EBP. Particular emphasis was placed on their ability to synthesise valid evidence, lead the implementation process effectively and evaluate its outcomes. According to the respondents, strengthening this area of competence requires a clearer definition of nurse managers' roles and expectations in implementation to promote more consistent and systematic EBP following the organisational change.

But then, of course, these clinical guidelines and recommendations can often be quite challenging—especially if the nurse manager is left alone. You receive a guideline, and then you're expected to figure out how to implement it in your own unit, and that can be really difficult. P5

#### 5.4.2 Digital Information Systems

According to the clinical nurse specialists and nursing directors, a key enabler for nurse managers in leading EBP was the use of coherent digital information systems that provided measurable values of quality and effectiveness of care.

The clinical nurse specialists and nursing directors stated that due to the organisational change, the lack of integrated information systems hindered the ability of nurse managers to lead EBP. The respondents highlighted that measuring the quality of care achieved through evidence‐based practice was essential. They emphasised the importance of quality management platforms in assessing the quality of evidence‐based interventions and in identifying suitable indicators for evaluating the health benefits for patients. Both clinical nurse specialists and nursing directors noted that the collected data should include qualitative insights in addition to quantitative or numerical data.

In our patient satisfaction surveys, we don't really ask, ‘Did you think that the interventions you received were useful just for you?’ They are more about overall patient satisfaction. P6

The respondents emphasised the importance of nurse managers being able to access, analyse and interpret the information they need, as well as having time, resources and advanced tools to lead EBP. Organisational change highlighted the need to identify consistent and accurate measures, such as determining what information to track and where to obtain it, to measure the effectiveness of care. The consolidation of various departments had left the organisation with multiple patient and other information systems that did not yet support nurse managers in leading EBP. The respondents recognised that different areas of the organisation had unique demands.

Right now, at least in our wellbeing services county, we're still trying to figure out what indicators we're tracking, what kind of data we want, and where we want it from. And then evaluating the impact of all these actions—that's really challenging, because it takes time, and finding the right indicators is not easy. P4

## 6 Discussion

This study identified enablers and barriers to nurse managers' leadership of EBP during organisational change from the perspectives of clinical nurse specialists and nursing directors. We found three main categories: (1) organisational readiness, (2) partnerships and (3) expertise in evidence‐based practice. Organisational readiness included standardised practices and supportive organisational structures. Partnerships were enabled through clinical nurse specialists, interprofessional collaboration and management support. Expertise encompassed nurse managers' competence and the use of digital systems to evaluate care quality and effectiveness.

The organisation under study demonstrated unequal opportunities for nurse managers to lead EBP due to insufficient organisational readiness during the change. Readiness—including leadership support, clear goals and organisational culture—forms the foundation for successful implementation (Vaishnavi et al. 2019). Embedding EBP into the organisational culture and supporting current and future leaders are essential strategies (Cleary‐Holdforth et al. 2022). The importance of organisational culture during change is also supported by Frilund et al. (2023). An evidence‐based practice culture, strengthened by mentorship, positively influences nurses' competence, job satisfaction and retention, thereby supporting nurse managers in their leadership roles (Melnyk et al. 2021). The findings revealed that clearly defined roles and standardised practices support nurse managers in leading EBP. However, organisational change increased their administrative workload, particularly in human resource management, limiting their ability to focus on leading EBP. Time constraints, lack of resources and high workload have been identified as key barriers to implementation (Harvey et al. 2020). The change process also exposed structural inequalities, especially between specialised and primary care, and in the uneven availability of clinical nurse specialists. Strengthening organisational support—particularly through adequate staffing, human resources assistance and clear communication—is essential to enable nurse managers to lead evidence‐based practice effectively (Bianchi et al. 2018).

According to the clinical nurse specialists and nursing directors, partnerships with clinical nurse specialists, interprofessional collaboration and managerial support empower nurse managers to lead EBP effectively during organisational change. Our study aligns with Crawford et al. (2023), who emphasise pragmatic solutions for building evidence‐based practice capacity, requiring frontline nurse feedback, commitment and partnership with nursing leaders. Pate and Rankin (2022) also emphasise that collaboration between clinical nurse specialists and clinical nurse supervisors enhances evidence‐based practice implementation and improves nursing‐sensitive outcomes such as infection rates, falls and patient satisfaction. Furthermore, our findings highlight the need for multilevel national collaboration to support consistency in leadership of EBP. In Finland, the Nursing Research Foundation (NRF 2024) has developed tools such as the FinAME and OMEBP models to guide the implementation of EBPs. These models help nurse managers apply structured, clearly defined approaches, particularly during organisational change. In addition, our study underscores the importance of emotional and practical support from management, especially for newly appointed nurse managers or those transitioning to new roles. Such support has been shown to improve their experiences during major organisational transformations (Morrison and Jensen 2022).

Our results indicate that leading EBP during organisational change requires a clear understanding of the nurse manager's role. The respondents in our study highlighted that while theoretical education from universities provides foundational knowledge, practical experience is essential for translating this into effective leadership. These findings align with Ominyi et al. (2025), who note the complex responsibilities nurse managers face in balancing clinical and managerial roles within constrained organisational environments. To support managers, integrating evidence‐based practice competencies into job descriptions and clinical promotion systems may clarify expectations and incentivise performance (Melnyk et al. 2016).

Fragmented and poorly integrated digital systems hinder nurse managers' ability to lead EBP by limiting access to current guidelines and reliable patient data, and by complicating outcome evaluation due to inconsistent records, as perceived by the clinical nurse specialists and nursing directors. The respondents emphasised the need for more advanced, unified systems and consistent indicators across regions to enable meaningful evaluation of care quality and outcomes. Such systems would improve comparability of data and strengthen nurse managers' ability to guide evidence‐based care. The critical role of digital technology in supporting real‐time access to evidence, clinical decision‐making and ongoing training has been highlighted by Ominyi et al. (2025). Updated, research‐based guidelines and protocols within digital systems also help ensure adherence to best practices.

Proactively anticipating change and involving key stakeholders in the planning phase can foster an environment more conducive to successful implementation. Several change theories, including those by Pettigrew et al. (1992) and Weiner (2009), emphasise the significance of organisational context, staff involvement and adaptability throughout the change process. To support EBP, organisations must ensure structural support, professional competence and systematic monitoring of care outcomes to guide improvements. According to Weiner (2009, 2020), organisational readiness for change is shaped by members' shared commitment to change and their collective belief in their ability to implement it—referred to as change efficacy. Readiness is influenced by how members value the change and how they assess task demands, resource availability and situational factors. High readiness enhances the likelihood of successful change by promoting effort, persistence and collaboration. When organisational readiness is high, members are more likely to initiate and sustain change efforts, persist through challenges and actively support implementation (Weiner 2020). Nurse managers' ability to lead evidence‐based practice during organisational change is not solely dependent on their individual competence or motivation, but is strongly influenced by the organisation's readiness—namely, its commitment, collective confidence, supporting structures and leadership alignment.

Updating the substance of leadership training to ensure it provides sufficient theoretical knowledge for leading evidence‐based practice is essential. Our study showed that, in addition to theoretical knowledge, practical leadership skills are also needed to effectively guide evidence‐based practice. A study by Koivunen et al. (2024) highlights the limited understanding of nurse managers' competence in leading EBP. To ensure high‐quality care, it is essential to strengthen their skills through updated and targeted education. Nurse managers must develop both theoretical and practical evidence‐based practice competencies to effectively support direct care nurses and act as role models. Developing valid tools to assess these competencies is also crucial for guiding future training and leadership development.

As perceived by the clinical nurse specialists and nursing directors, nurse managers can influence change commitment by reinforcing the perceived value and relevance of EBP and aligning them with organisational goals. Their leadership is more effective when they are supported by clear structures, shared practices and collaborative partnerships across professions. Interprofessional collaboration and management support enhance nurse managers' motivation and engagement, particularly in uncertain or rapidly evolving situations. Change efficacy can be strengthened by ensuring nurse managers receive appropriate training, supervisory support, adequate resources and access to functional systems for evaluating care outcomes. These elements are essential for enabling nurse managers to lead EBP in ways that directly improve the quality and effectiveness of care.

### 6.1 Strengths and Limitations of the Work

This study provides valuable insights into nurse managers' ability to lead EBP during organisational change, as the informants, clinical nurse specialists and nursing directors, were well‐acquainted with the organisation and its practices. However, this study has several limitations. One limitation is that it was conducted in a single wellbeing services county in Finland. As a result, the findings may not be transferable to all international contexts.

The perspectives on enablers and barriers for nurse managers in leading EBP during organisational change were collected from clinical nurse specialists and nursing directors rather than from the nurse managers themselves. This approach was chosen to gain a broader, strategic‐level understanding of the organisational conditions, leadership expectations, and system‐level enablers and barriers related to leading EBP. While this may be considered a limitation in terms of not capturing the nurse managers' own experiences, it provided valuable insight into the organisational readiness and leadership structures influencing evidence‐based practice during change. Our respondents were closely connected to the nurse managers and had the opportunity to observe their work up close. They also experienced the organisational change themselves and were experts in their respective areas. Nevertheless, because data represent second‐order interpretations, some interpretive distance may remain and should be considered when interpreting the findings.

### 6.2 Recommendations for Further Research

This study highlights several important options for future research on nurse managers' leadership of evidence‐based practice. It would be valuable to explore nurse managers' perspectives on how organisational change affects the leadership of evidence‐based practice. Multi‐organisational case studies could offer insight into how different organisational cultures shape leadership practices during times of transition. Longitudinal studies are needed to examine whether the identified enablers and barriers to evidence‐based practice are sustained, evolve or diminish over time.

Targeted interventions—such as mentoring programmes or data dashboards—should be piloted to assess their impact on evidence utilisation and patient outcomes. Further research should also investigate how leadership decisions are reflected in everyday clinical practice, and how patients and family caregivers perceive the influence of leadership on evidence‐based practice on the quality and safety of care.

Comparative studies between professional groups, such as leaders in nursing and medicine, could help identify shared and distinct approaches to leadership of evidence‐based practice. The role of digital infrastructure—including electronic health records and clinical decision support systems—should be examined to support the real‐time use of evidence. Additionally, policy‐level factors such as financial incentives and accreditation requirements warrant investigation for their role in enabling or constraining leadership of evidence‐based practice.

Finally, future studies should explore the developmental pathways of leadership education and their influence on the acquisition and strengthening of evidence‐based leadership competencies among nurse leaders. Longitudinal, multi‐site and intervention‐based research is essential to deepen understanding of how leadership of evidence‐based practice can be supported in evolving organisational contexts.

## 7 Conclusion

This study identified key enablers and barriers to nurse managers' leadership of EBP during organisational change, based on perspectives from clinical nurse specialists and nursing directors. Three main categories emerged: (1) organisational readiness, (2) partnerships and (3) expertise in evidence‐based practice. Our findings indicate that organisational change significantly affects nurse managers' ability to lead EBP. Strengthening change readiness, building partnerships and developing the leadership of evidence‐based practice are essential for ensuring the quality and effectiveness of nursing care during organisational change. The findings of this study are important for patients, staff and the organisation, as they highlight areas that should be addressed proactively—both before organisational change and during the planning of new operations. This study demonstrates that organisational readiness, partnership and expertise in evidence‐based practice are key factors in determining the success of nurse managers' leadership of evidence‐based practice during organisational change.

## Author Contributions

**Riitta Askola:** conceptualization, methodology, investigation, formal analysis, writing – original draft, writing – review and editing. **Anna Axelin:** conceptualization, formal analysis, supervision, writing – review and editing. **Laura‐Maria Peltonen:** conceptualization, writing – review and editing. **Miia Lindström:** conceptualization, writing – review and editing. **Jaakko Varpula:** conceptualization, methodology, investigation, writing – review and editing. **Tarja Heino‐Tolonen:** conceptualization, writing – review and editing.

## Funding

This work was supported by Stiftelsen Eschnerska Frilasarettet sr.

## Ethics Statement

The study received ethical approval from Ethics Committee for Human Sciences at the University of Turku, Health Care Division (TY/461/06.01.01/2024), and research permission was obtained from the study organisation.

## Consent

Participation was voluntary, and informed consent was obtained from all participants. Audio recordings were deleted after transcription to ensure data confidentiality.

## Conflicts of Interest

The authors declare no conflicts of interest.

## Data Availability

Data sharing not applicable to this article as no datasets were generated or analysed during the current study.
